# Osteocytes but not osteoblasts directly build mineralized bone structures

**DOI:** 10.7150/ijbs.61012

**Published:** 2021-06-11

**Authors:** Ke Wang, Yinshi Ren, Shuxian Lin, Yan Jing, Chi Ma, Jun Wang, X. Baozhi Yuan, Xianglong Han, Hu Zhao, Zheng Wang, Minghao Zheng, Yin Xiao, Lin Chen, Bjorn Reino Olsen, Jian Q. Feng

**Affiliations:** 1 Department of Biomedical Sciences, Texas A&M University College of Dentistry, Dallas, TX 75246, USA.; 2 Center for Excellence in Hip Disorders, Texas Scottish Rite Hospital for Children, Dallas, TX 75219 USA.; 3 Laboratory of Oral Biomedical Science and Translational Medicine, School of Stomatology, Tongji University, Shanghai, 200092, China.; 4 Department of Orthodontics, Texas A&M University College of Dentistry, Dallas, TX 75246, USA.; 5 State Key Laboratory of Oral Diseases, National Clinical Research Center for Oral Diseases, West China Hospital of Stomatology, Sichuan University, Chengdu, 610041, China.; 6 Angitia Biopharmaceuticals, Guangzhou, 510000, China.; 7 Department of Restorative Dentistry, Texas A&M University College of Dentistry, Dallas, TX 75246, USA.; 8 Centre for Orthopaedic Research, School of Surgery, The University of Western Australia, Perth, 6009, Australia.; 9 Institute of Health and Biomedical Innovation, Queensland University of Technology, Brisbane, Queensland, 4059, Australia.; 10 Department of Rehabilitation Medicine, Center of Bone Metabolism and Repair, State Key Laboratory of Trauma, Burn and Combined Injury, Daping Hospital, Third Military Medical University, Chongqing, 400042, China.; 11 Department of Developmental Biology, Harvard School of Dental Medicine, Boston, MA 02115, USA.

**Keywords:** Mineralization, osteocyte, osteoblast, DMP1, Phex, bone formation, hypophosphatemia rickets

## Abstract

Bone-forming osteoblasts have been a cornerstone of bone biology for more than a century. Most research toward bone biology and bone diseases center on osteoblasts. Overlooked are the 90% of bone cells, called osteocytes. This study aims to test the hypothesis that osteocytes but not osteoblasts directly build mineralized bone structures, and that defects in osteocytes lead to the onset of hypophosphatemia rickets. The hypothesis was tested by developing and modifying multiple imaging techniques, including both *in vivo* and *in vitro* models plus two types of hypophosphatemia rickets models (*Dmp1*-null and Hyp, Phex mutation mice), and *Dmp1*-Cre induced high level of β-catenin models. Our key findings were that osteocytes (not osteoblasts) build bone similar to the construction of a high-rise building, with a wire mesh frame (i.e., osteocyte dendrites) and cement (mineral matrices secreted from osteocytes), which is a lengthy and slow process whose mineralization direction is from the inside toward the outside. When osteoblasts fail to differentiate into osteocytes but remain highly active in *Dmp*-1-null or Hyp mice, aberrant and poor bone mineralization occurs, caused by a sharp increase in Wnt-β-catenin signaling. Further, the constitutive expression of β-catenin in osteocytes recaptures a similar osteomalacia phenotype as shown in *Dmp1* null or *Hyp* mice. Thus, we conclude that osteocytes directly build bone, and osteoblasts with a short life span serve as a precursor to osteocytes, which challenges the existing dogma.

## Introduction

Bone growth and formation is a long, complex process 1. Currently, mostly all bone studies are focused on the balance of osteoblast (Ob) and osteoclast functions on bone surfaces 2-4. For a long time, the early assumptions on Obs forming bone could not be directly tested due to the lack of proper technology. Ironically, it is extremely difficult to envision how cells with a short lifespan in bone surface layers directly form into well-built 3D deep bone structures (similar to a building process without builders working inside of the building).

The roles of osteocytes (Ocys), as the most abundant bone cells with large cellular surfaces and half-lives of decades 5, remain speculative even though their locations would be ideal for carrying out this job. Recent studies indicate that Ocys are more active than previously thought 6; they play a role in the regulation of osteoclast formation 7, 8 and mechanical strain reactions 9, 10. They participate in bone mineralization 11, 12; and they are involved in bone mineral removal during lactation 13. They coordinate adaptive bone remodeling 14 and regulate phosphate homeostasis 11. It has been known that bone is filled with an Ocy-lacuna-canalicular system 15, in which Ocys demineralize them to keep a low mineralized zone along this system for a connection to vessel systems 13, 16 and to keep interstitial fluid flow 17.

There are different types of rickets, but all are due to poor mineralization with either low calcium and/or phosphate 18-20. Although it has been suggested that Ocys play a critical role in the phenotypes observed in mice with DMP1 mutation mice, and *Dmp1-*null mice 11, defects of Obs are still believed to be responsible for the onset of bone defects.

In this study, we address three key unsolved issues in basic bone biology and the pathological changes in hypophosphatemia rickets: 1) the cell directly responsible for building bone structure and mineralization, 2) the mineralization direction (from outside to inside or from inside toward the outside) and 3) the causative factor initiating the onset of mineralization defects in rickets. The experimental approach includes novel combinations of multiple imaging techniques with two genetically engineered rickets models plus the constitutive stabilization of β-catenin in Ocys for mechanism studies. Our studies demonstrate that Ocys (not Obs) build bone similar to the construction of a high-rise building with a wire mesh frame (Ocy dendrites) and cement (mineral matrices secreted from Ocys); when Obs fail to differentiate into Ocys in *Dmp*-1-null or Hyp (a Phex mutation) mice during development, aberrant bone mineralization occurs due to a sharp increase in Wnt-β-catenin signaling, resulting in rickets.

## Materials and Methods

### Mice

All procedures involving animals were performed in accordance with protocols approved by the Institutional Animal Care and Use Committee (IACUC) at Texas A&M University College of Dentistry. Mouse *Dmp1* knockout (KO) 21, *Dmp1*-Cre 22, and 2.3 Col-GFP 23 mice were on a mixed C57BL and CD-1 background; *β-Catenin^flox(ex3)^*loxP 24 and Hyp mice (stock number: 000528, the Jackson Laboratory) were on a C57BL background. Mice were maintained in an animal facility on a 12-hr light/dark cycle at an ambient temperature of 20-22°C. All experiments on mice were of mixed sexes.

### X-ray

Bone samples were taken for X-ray images using the Faxitron MX-20 Cabinet X-ray System (Faxitron X-Ray LLC, Lincolnshire, IL, USA).

### Tissue Preparation

Long bone and mandible samples were collected from the animals and fixed in 4% paraformaldehyde in phosphate-buffered saline (PBS, pH = 7.4) at 4°C for 2-4 days. Nondecalcified tibias were dehydrated in ascending graded ethanol (EtOH) (75%, 95%, and 100% twice, 2-4 days each) followed by xylene and embedded in methyl-methacrylate (MMA, Buehler, Lake Bluff, IL) as previously described 25. The other side of the long bones were decalcified in 15% EDTA at 4°C, embedded in paraffin, and cut into 4 µm-thick sections 26. For TEM studies, the samples were processed in a special fixation fluid (2% glutaraldehyde and 2% paraformaldehyde in 0.1M buffer) as previously described 27.

### Transmission electron microscopy (TEM), backscattered and acid-etched scanning electron microscopy (SEM), and Energy Dispersive X-ray (EDX) Microanalysis

For TEM images, the non-decalcified thin bone sections were cut and stained with uranyl acetate and lead citrate, then examined using a Philips CM12 in TEM mode as previously described 27.

Methylmethacrylate (MMA) embedded tibia blocks were used for SEM analyses as previously described 27. Briefly, the surfaces of MMA-embedded blocks were polished using 800 grit and 1200 grit sandpaper sheets (Buehler), followed by 1-, 0.3-, and 0.05 μm MicroPolish II alumina solutions (Buehler) on a rotating wheel covered with a soft cloth. The blocks were cleaned in an ultrasonic bath and air-dried in a vacuum for 48 hours. The dehydrated samples were carbon coated and imaged with backscattered SEM (JEOL JSM-6010LA, Japan). EDX was measured to analyze differences in mineral content from various anatomic locations in mice.

For resin-casted osteocyte-lacuno-canalicular SEM, the surfaces of MMA-embedded bones were re-polished and acid-etched with 37% phosphoric acid for 2-10 seconds, followed by two 20-minute washes with 5% sodium hypochlorite. Subsequently, the blocks were washed, dried, and coated with gold/palladium prior to SEM imaging 28.

### VolumeScope 29

The 2-month-old decalcified WT and *Dmp1* KO tibia samples were trimmed using a diamond wire saw. The trimmed blocks were then glued on an SEM stub (Agar Scientific, AGG1092450) using a two-component silver conductive epoxy H20E EPO-TEK (Tedpella). The samples were finally trimmed down to a block face of 350 µm x 250 µm and 200 µm deep using an ultramicrotome. To minimize charging of the block when imaged with the electron beam, the bottom and sides of the block were sputter coated with a 30nm thick layer of gold.

The samples were then imaged using an Apreo scanning electron microscope (Thermo Fisher Scientific, Waltham, USA). The microscope was equipped with a VolumeScope module for Serial Block face Imaging (SBFI), operating in low vacuum mode at 50 Pa using a lens mounted backscattered detector.

The dataset was collected using an accelerating voltage of 2kV and a beam current of 100pA. A total of 295 images from each sample were collected using a 40 nm slice thickness. Dimensions of the images were 8192 x 8192 with a pixel resolution of 10nm in x and y, using a dwell time of 1 µs.

### Fluorochrome Labeling

To analyze the role of osteocytes in mineralization, wild-type mice were injected 5 days apart with calcein (5 mg/kg i.p, 1^st^ injection) and alizarin red (20 mg/kg i.p, 2^nd^ injection) as previously described 27. Thick sections (300-400 μm) were cut from the MMA-embedded blocks of these animals with a diamond-bladed saw (Buehler, Lake Bluff, IL), ground down to a final thickness of 30-50 μm, and polished. Fluorochrome labeling was then photographed using a Leica TCS SP5-II upright microscope combined with or without DAPI staining of nuclei of osteocytes 30.

For double mineral labeling, adult mice were i.p. injected with Calcein (5 mg/kg) and Alizarin Red (20 mg/kg) 5 days apart, with mice sacrificed two days after the second injection as previously described 27. In the experiment with the P8 2.3 Col1-GFP mouse model, alizarin red (20 mg/kg i.p) was injected at 5 different time periods from 5 min, 15 min, 30 min, 60 min, and 4 hours before harvest to demonstrate the short-term effect of mineral deposition by osteocytes. Nondecalcified tibia samples were embedded in optimum cutting temperature compound (O.C.T. compound, Tissue-Tek) and cut into 10-μm-thick frozen sections using a transparent film as previously described 31, followed by confocal imaging.

### Fluorescein Isothiocyanate (FITC) Stain

The osteocyte-canalicular system was visualized by FITC (a small molecular dye) which fills in all the cells, dendrites, and blood vessels (but will not enter the mineralized matrix), followed by an MMA embedding method as previously described 32. Thus, the dye provides a visual representation for the organization of the osteocyte-canalicular system and blood vessels under the confocal microscope. Cross-sections with around 1 mm thickness were cut with a diamond-bladed saw (Buehler, Lake Bluff, IL) and the sections were then sand grounded to a final thickness of ~50 μm for confocal images. Osteocyte morphometry was quantified using Imaris software 32.

### Histology and Immunohistochemistry

Nondecalcified MMA-embedded blocks were cut into 5 µm-thick sections (Leica Polycut S microtome) for McNeal von Kossa stains and Masson-Goldner trichrome stains 33. Decalcified paraffin sections were used for H&E staining and Sirius red staining as previously described 28, 34, 35. Immunohistochemistry and immunofluorescence were performed as previously reported 36.

### *In Situ* Hybridization

Digoxigenin (DIG)-labeled cRNA probes were prepared by using the RNA Labeling Kit (Roche, Indianapolis, IN, USA). The cDNA probes for Tcf1, Runx2, Osx, and osteocalcin (OC) were gifts from Dr. Stephen E Harris, University of Texas Health Science Center at San Antonio that were lineaged as previously described 37. The hybridization temperature was set at 55°C and the washing temperature was at 70°C, which inactivates endogenous AP. DIG-labeled nucleic acids were detected in an enzyme-linked immunoassay with a specific anti-DIG-AP antibody conjugate and the color substrates 4-nitro blue tetrazolium (NBT) and 5-bromo-4-chloro-3- indolyl phosphate (BCIP) according to instructions from the manufacturer (Roche). The hybridization signals were photographed with a Nikon E800 microscope (Nikon, Tokyo, Japan).

### Real Time RT-PCR Assay

The extracted RNAs were used as templates for reverse transcription PCR using a SuperScript III reverse transcription kit (Invitrogen, CA, USA). The cycling protocol was as follows: 95 °C for 10 min, 40 cycles of 95 °C for 15 s, and 60 °C for 1 min. TaqMan Universal PCR mix and TaqMan probes specific to Ctnnb1 (Mm 00483039_ m1) were used for real-time PCR. The real-Time PCR was performed using 7500 Fast Real-Time PCR Software 1.3.1 (Applied Biosystems, CA, USA). Gapdh (Mm99999915_g1) and GAPDH (Hs 02786624_ g1) (Applied Biosystems, CA, USA) were used as the endogenous control. All reactions were run in triplicates.

### Western Blot

For preparation of Western blots, a 20-µg aliquot of the protein extracts was electrophoresed in 10% SDS-PAGE gel and transferred to a nitrocellulose membrane. The membrane was washed and probed with β-catenin 71-2700 (Thermo Fisher Scientific, Massachusetts, USA). To quantify the immunoblotting signal, 6 ml of chemiluminescence detection solution (ECL Plus; Amersham, Van Nuys, CA) was added and the signal was detected by a fluorescence scanner (Storm 860; Amersham, Van Nuys, CA). The density of bands was analyzed using ImageQuant 5.2 software (Molecular Dynamics, Sunnyvale, CA). The antibodies were subsequently stripped off by incubation in reprobing solution (62.5 mM Tris-HCL, 2% SDS, and 100 mM 2-mercaptomethanol, pH 6.7) for 30 minutes at 50°C. The membrane was then blocked and probed with β-actin antibody (for the remaining proteins) to verify the loading equivalence among samples.

### Murine Primary Osteoblast Culture

Mouse calvarial primary osteoblasts were isolated from 4-day-old heterozygous DMP1-lacZ knock-in mice by sequential trypsin and collagenase digestion as previously described 38. These cells were plated into 6 cm petri dishes together with the bone chips in α-MEM supplemented with 10% fetal bovine serum (FBS) and 100 U/ml penicillin/streptomycin (P/S). At confluence, the cells were digested with trypsin and seeded into 24-well-plates at 5 x 10^4^ cells/cm^2^ in α-MEM supplemented with 10% FBS, 100 U/ml P/S, 10 mM β-glycerophosphate (β-GP), 50 ug/ml ascorbic acid, and 10 nM dexamethasone. They were cultured for 14 days with Alizarin Red (0.1%; for staining mineralized nodules). At the end of the culturing, plates were fixed in 0.5 % glutaraldehyde for 10 min on ice followed by X-Gal staining for expression of lacZ in these cultured cells. The images were captured under a Nikon E800 microscope with MetaMorph software.

### Quantitation and Statistical Analyses

Data were analyzed with SPSS software (version 16.0; SPSS, Chicago, IL). All results were presented as mean ± standard deviation. A two-tailed, independent student t-test was used to evaluate the differences between control and Dmp1 KO groups (* p < 0.05, ** p < 0.01, *** p < 0.001).

## Results

### Bone Is Built with A “Rod and Wire Mesh” by Osteocytes and Their Dendrites

**Figure 1:**
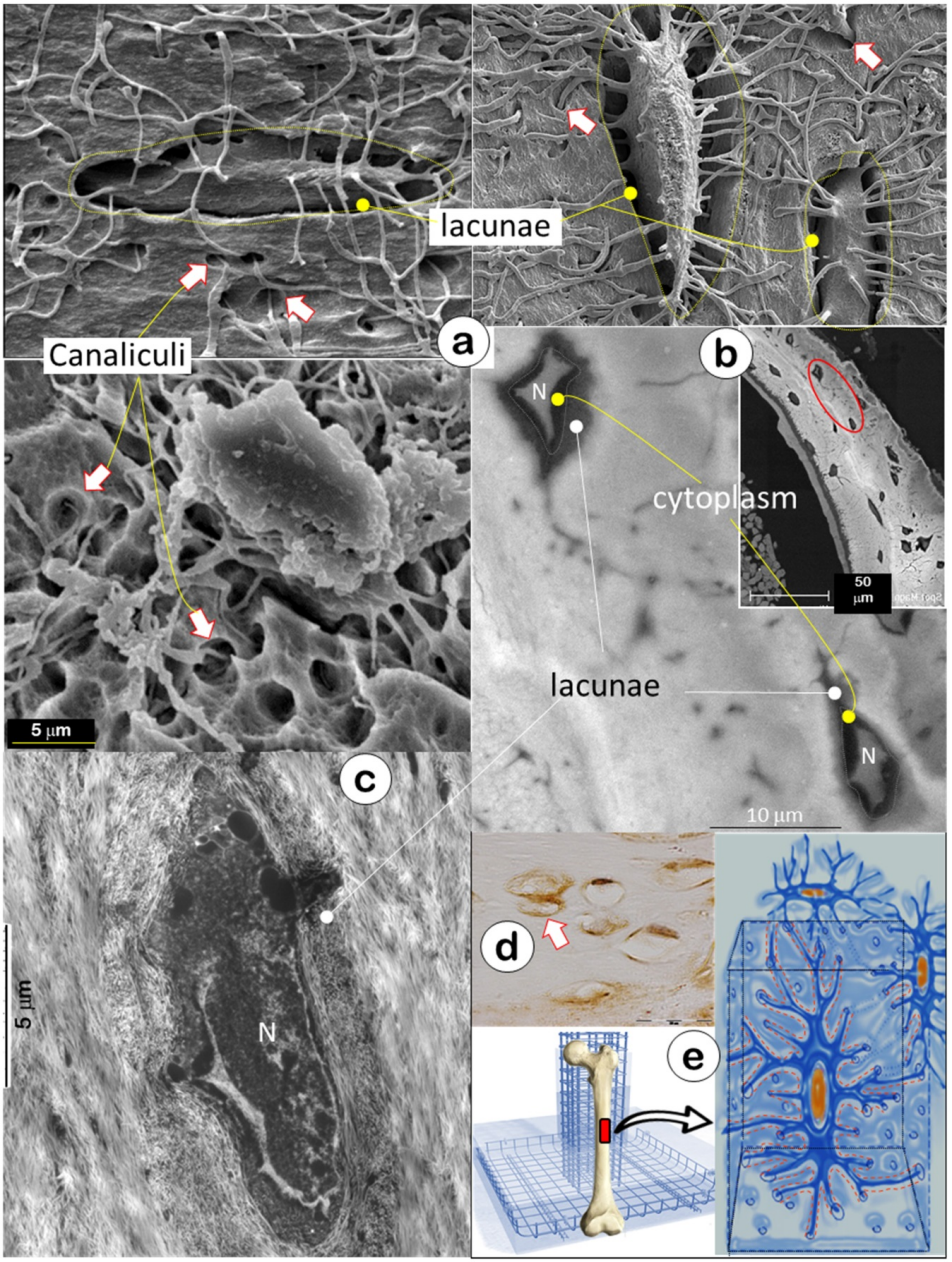
** Bone is constructed with a special “wire mesh”: osteocytes and their dendrites** (Representative data from 2-4 animals for each assay)**.** (a) Acid-etched SEM images from mouse bones revealed a “rod and wire mesh-like” structure, in which osteocytes work as a rod and their dendrites behave as mesh in all bones tested, including calvaria (upper left), mandible (lower), and long bones (upper right) (n = 4). (b) The backscattered TEM image showed fibers in lacunae that differ from the classic collagens in bone matrices, including distribution patterns and fiber size (N=nucleus) (n = 2). (c) The TEM image displayed a grey color in the lacunae area (n = 3). (d) The immunostain showed high levels of biglycan in the lacunae region (n = 3). (e) The working hypothesis was that the osteocyte-dendrite structure is similar to a rod and wire mesh building structure.

The skeleton is composed of a few hundred different types of bone. In this study, we used acid-etched SEM (a technique removing surface minerals 11) to expose inside bone structures of calvarias (**Figure** 1a, *upper left*), mandibles (**Figure** 1a, *lower*), and long bones (**Figure** 1a, *upper right*), respectively. These SEM images showed empty spaces (i.e., hypomineralization zones with their low mineral contents removed by acid treatment which has little impact on deep bone matrices) surrounding lacunae and canaliculi created by acid removal of surface minerals and matrix tissue (arrows); this finding suggested that bone is essentially built with a special “wire mesh frame”. The backscattered SEM image further confirmed a low mineral content in the lacunae as reflected by the grey color in contrast to a high mineral layer on its outside (white mineralization rings, **Figure** 1b). The TEM image showed that the fibers in the low mineral zone were thin with a different distribution pattern from outside type I collagen, the main fibers for calcium and phosphate ion binding and subsequent nucleation of apatite crystals 39 (**Figure** 1c). The immunostain displayed a high content of biglycan (an inhibitor of mineral growth 40, 41) in the lacunae (**Figure** 1d, arrows), which help maintain the lacuna-canalicular system in a low mineral zone. The “rod and wire mesh-like” structure was also observed in the surface bone from a 42-year-old mandibular cadaver, although the deep mature bone cannot be exposed due to a bounded high mineral construct by a surface acid-etched technique (**Figure** S1). Thus, we propose that the Ocy-lacuna-canalicular system, as a special rod and wire mesh-like structure, constructs the bone frame (**Figure** 1e) for filling with “cement” (minerals; see below for details).

### The Direct Association between the Unique Structure of Osteocytes and the Slow, Lengthy Bone Mineralization Process

**Figure 2:**
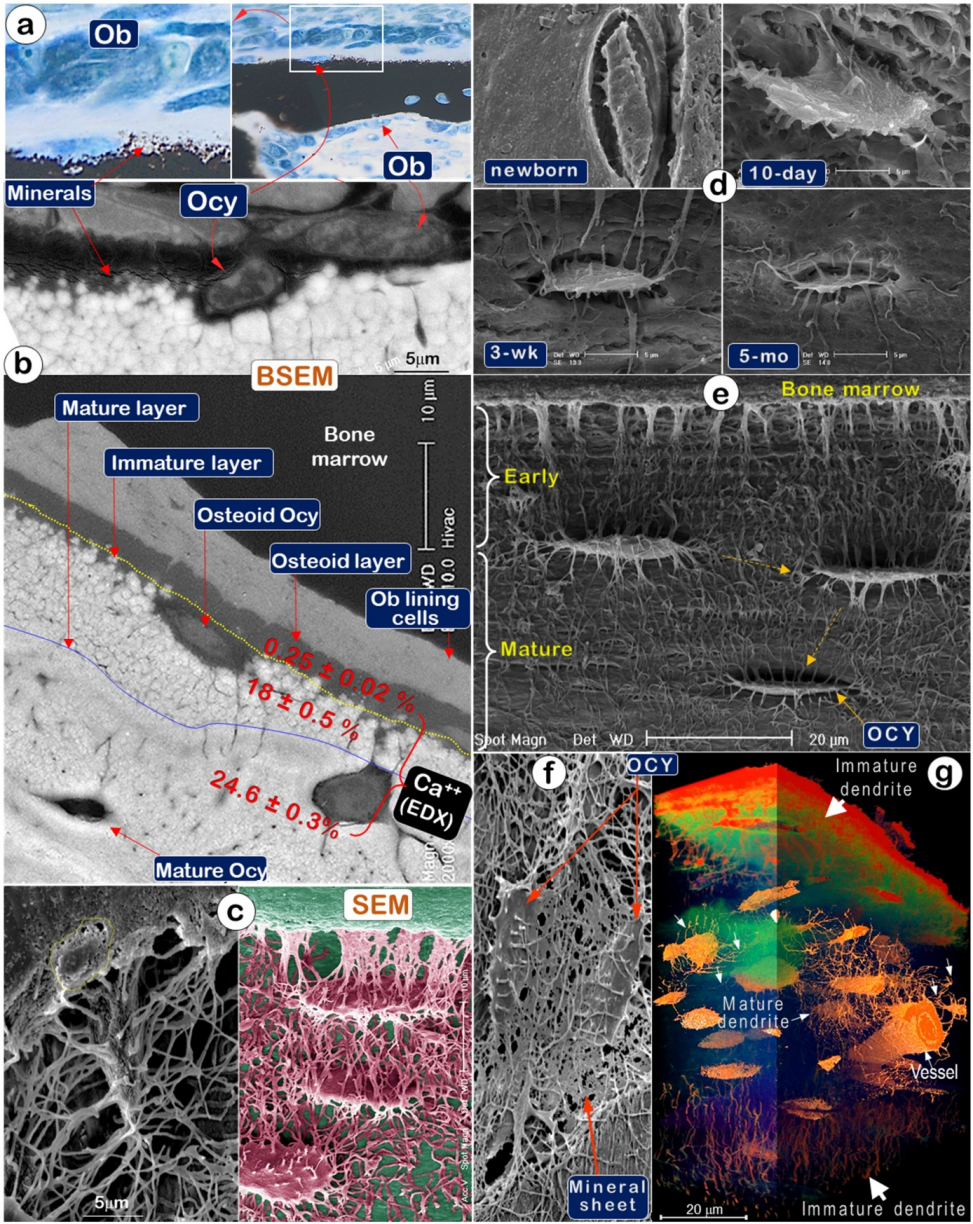
** Mineralization is directly linked to osteocytes (Ocys) but not osteoblasts (Obs)** (Representative data from 2-5 animals for each assay). (a). Von Kossa staining showed newly formed mineral particles surrounding an Ocy site, far away from the Ob layers in the periosteum (n = 5); (b). Backscattered SEM (BSEM) image of the same bone exhibited (n = 3): lack of minerals in osteoblast layer; low mineral levels in the osteoid-layer, as indicated by calcium content (Ca^2+^) of 0.25±0.02% by EDX; a poorly formed mineral layer with individual sphere-like mineral structures (Ca^2+^, 18±0.5%); a mature mineral-layer (Ca^2+^, 24.6±0.3%), in which Ocys were surrounded by dense mineralized rings, suggesting that mineralization is initiated from Ocys and not Obs. (c). The acid-etched SEM images of the osteoid layer in a 2-month-old mouse femur displayed numerous Ocy dendrites surrounded by many empty spaces created by acid removal of mineralized matrix. An Ob is buried in the bone marrow filled with resin (n = 4). (d). Acid-etched SEM images of murine calvaria revealed an absence of minerals and very few Ocy dendrites in newborns (upper left), poor mineralization, and inadequate Ocy maturation in 10-day-old mice (upper right), whereas calvaria at 3 weeks (lower left) and 5 months of ages (lower right) display resistance to acid-etching due to enhanced matrix mineralization (n = 1). (e). The acid-etched SEM image of a 2-month-old mouse long bone showed poor mineralization in the early layer, which progresses to well-established mineralization and Ocy maturity (spindle-shaped cells with reduced volume) (n = 4). (f). The acid-etched SEM image of a 2-month-old mouse mandible displayed “thin mineral sheets” directly linked to Ocy dendrites (n = 1). (g) VolumeScope 3D imaging data showed immature dendrites (thick and short) of Ocys close to bone surfaces and mature dendrites (thin and long) of Ocys in deep bone areas (n =2).

To test that Ocys are essential for and play a prominent role in the production of construction cement (bone minerals), we first used Von Kossa staining (a classic mineralization assay) and showed newly formed mineral particles surrounding Ocys but far away from Ob layers (**Figure** 2a). Backscattered SEM (BSEM) images further confirmed this distribution pattern (**Figure** 2b, *upper*). Noticeably, BSEM revealed a mineral gradient (reflected by color changes) from none in bone marrow (black) to low levels in the osteoid layer (grey), then high levels in the immature mineral layer (in a white sphere-like nodular structure), and finally, the mature layer (in smoothly fused white matrices; **Figure** 2b,* lower*). EDX (Energy Dispersive X-ray spectroscopy) showed extremely low calcium content in the osteoid layer, the site of initial mineralization by Ocys (0.25±0.02%); there was increased calcium content in the immature mineral layer (18±0.5%, in which mineral particles remain separately), and a further increase in the mineral content of the mature matrix layer (24.6±0.3%), which contains mature Ocys and a homogeneous mineral structure (**Figure** 2b,* lower*; in which all mineral particles are fused together). Acid-etched SEM images of the osteoid layer on a murine long bone (2-month-old) displayed numerous Ocy dendrites surrounded by many empty spaces created by acid removal of mineral and matrix tissue, whereas an Ob cell marked with a yellow circle was buried in the bone marrow filled with resin, which could not be removed by acid treatment (**Figure** 2c, *left*). The artificially colored Ocy SEM image in the adjacent area showed a deep empty space close to bone marrows (reflecting a poorly formed mineral structure and the easy penetration of acid for removal of newly formed minerals). This image also showed solid matrices in the middle of the cortical bone (indicating a solid mineral content and the failure of acid to penetrate through the bonded mineral structures) (**Figure** 2c, *right*), supporting the direct relationship between Ocys (instead of Obs) and mineralization.

A temporal relationship between Ocy structure and mineralization in murine calvaria matrices was also demonstrated by experiments using an acid-etched SEM approach (**Figure** 2d). Specifically, the absence of mineralization in the newborn calvaria matrix was associated with the presence of nascent Ocys with few dendrites (**Figure** 2d, *upper left*). After 10 days, however, the appearance of plump young Ocys was associated with progressive mineralization in the calvarium (**Figure** 2d, *upper right*). By 3 weeks, the Ocys had transformed into spindle-shaped cells surrounded by a solid mineral matrix (**Figure** 2d, *lower left*). At 5 months, fully mature Ocys reduced to about 50% of their original body size and exhibited pericellular mineral growth (**Figure** 2d, *lower right*). Similarly, mineralization of murine femoral bones (2-month-old) was associated with Ocy maturation and a gradual reduction of the Ocy volume. The resulting vacant space created by the decrease in Ocy volume was filled by minerals, leading to increased bone mineralization (**Figure** 2e). The acid-etched SEM image also displayed a direct connection between Ocy dendrites and “mineral sheets” (**Figure** 2f). Additionally, the stacked series of ultrathin sections (40 nm/slide, a total of 295 slides) imaged by VolumeScope (a new imaging technique at the EM level) 29 provided a true 3D bone image. This image revealed a difference in Ocy morphologies between the newly formed ones on surfaces (thick diameter in dendrites) and the mature ones in deep bone (thin and well-defined dendrite structures) (**Figure** 2g).

### Osteocytes Are Essential for Producing Minerals *in Vivo* and *in Vitro*

**Figure 3:**
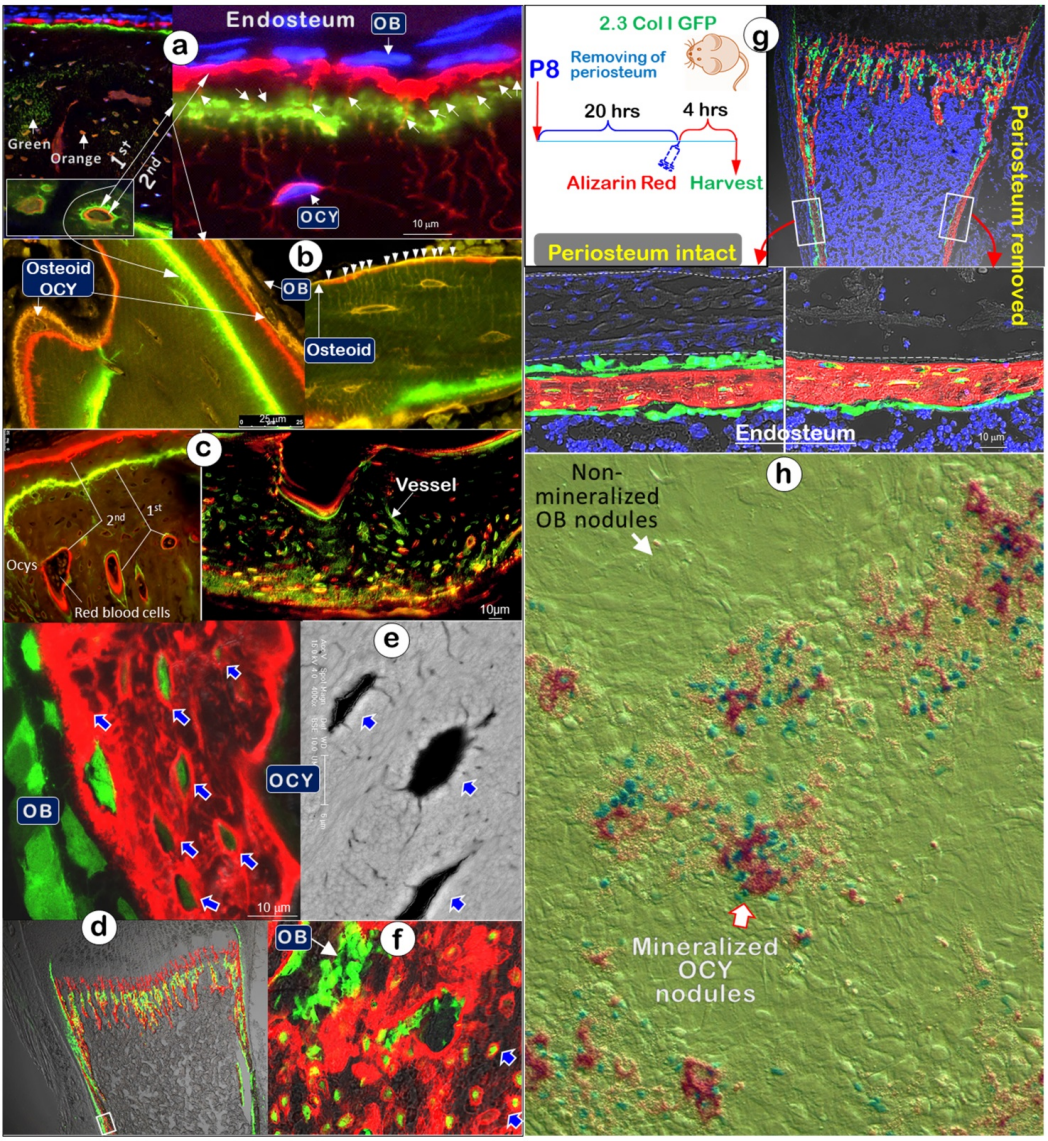
** Mineralization labeled “rings” are around osteocytes but not osteoblasts** (Representative data from 3-5 animals for each assay). (a). Fluorochrome-labeled images display 4 types of Ocys: green, orange (an overlap of green/red), green-red, and red-stained Ocy canaliculi (a lack of stained Obs) in cortical-bone (n = 3). (b). The mineral-labeled osteoid in trabecular bone was directly linked to Ocys (n = 4). (c). Mineral labeling around Ocys, the bone surface, and deep bone vessels in a 3-week-old mandible (left), and a 12-week-old humerus bone (right, cross-section) (n = 4). (d) Representative images from a P8 2.3 Col-GFP mouse 4 hours after Alizarin red injection revealed mineral deposition surrounding Ocys but not Obs (n = 4). (e). The backscattered-SEM showed strong mineral rings surrounding OCYs (n = 3). (f). Images from P8 2.3 Col-GFP calvarias with Alizarin red incorporated for 24 hrs. (n = 4). (g). Images from a P8 2.3 Col-GFP mouse with (right) and without (left) periosteum removal (for 20-hours) showed no apparent impact of periosteum removal on mineral depositions around Ocys (4-hour injection of Alizarin red; n = 4). (h). Calvarial cells isolated from 4-day-old heterozygous *Dmp1-lacZ* knock-in mice were cultured for nodule formation for 14 days, in which Alizarin red was added into the medium during culturing and the X-gal staining was performed at the end of experiment*.* Of note, the* lacZ* signal (green color) was associated with mineral content (red color). The white arrow points at non-mineralized nodules, the red-frame arrow points at the mineralized nodules, and the green cells indicate *Dmp1*-lacZ^+^ OCYs (n = 4).

To more precisely determine the functional role of Ocys in the mineralization process, we examined double-labeled fluorochrome specimens (5 days apart between Calcein, 1^st^ injection, and Alizarin Red, 2^nd^ injection, a gold standard for defining mineralization) isolated from the long bones of one-month-old mice using confocal microscopy. Sections from these bones with DAPI-stained cell nuclei revealed strong labeling lines directly linked to Ocys, with small amounts of fluorochrome-stained minerals surrounding the Ocy dendrites in the cortical areas (**Figure** 3a; **Figure** S1a; **Movie** S1). Of note, in mature Ocys deep into the bone, both Cacein and Alizarin Red labeling lines were clearly recognized, whereas only the late injected red labeling was seen in the newly formed Ocy cells at the surface. In contrast, no fluorescent label surrounded or was adjacent to the Obs at the bone surface, despite the widely held belief that Obs directly contribute to bone mineralization. In trabecular regions of long bones, Ocys were similarly labeled with different deposit patterns, including green Ocys (active mainly in the first labeling period) and yellow (active during the entire labeling period) (**Figure** 3b and **Figure** S1b). Importantly, the osteoid layer was an orange color (overlap of green and red labels), reflecting the contribution of mineral contents to bone surfaces from deep Ocy dendrites, in which green and red color layers failed to be separated (**Figure** S2). Furthermore, the double-labeled young mandible (**Figure** 3c, left) and adult humerus bone (**Figure** 3c, right) displayed an identical contribution of Ocys to mineral deposition around vessel areas.

To avoid missing any likely roles of Obs in early bone formation, we injected Alizarin Red into 8-day-old 2.3 Col 1-GFP (marking bone cells) mouse pups and harvested tissues after 5 min, 15 min, 30 min, 60 min (**Figure** S3a), and 4 hours (**Figure** 3d; arrows), respectively. Again, there was no red mineral deposition surrounding Obs (**Figure** 3d, **Figure** S4b-c). Noticeably, each GFP^+^ Ocy but not Ob was surrounded with a strong red labeling ring, similar to a strong white ring that surrounded each mature Ocy body in a backscattered SEM image (**Figure** 3e, arrows). To further support the *in vivo* studies, we cultured P8 2.3 Col 1-GFP calvarias *ex vivo* with low concentration of Alizarin Red integrated for 24 hours. The confocal image showed red incorporated rings surrounding Ocys but not Obs (**Figure** 3f).

In a separate experiment using 6-day-old 2.3 Col I-GFP mice, we removed the periosteal layer on one side of tibias and kept the other side intact for 20 hours, followed by a one-time injection of Alizarin red and sacrifice of the mice 4 hours later. The data showed little impact from removing the Ob-rich periosteal layer on the Ocy mineral deposition pattern (**Figure** 3g, arrows).

The *in vitro* mineral nodule assay 42-44 is a widely used approach to study the roles of Obs in mineralization. To better define cell contribution to mineralization, we developed a dual-alizarin red/X-gal stain assay using calvaria cells isolated from 4-day-old heterozygous *Dmp1*-lacZ knock-in mice, in which the lac Z is mainly expressed in Ocys 21. Alizarin Red was added during the entire nodule formation period to labeling minerals and X-gal was used to stain positive cells in the nodules at the end of cell cultures. Again, there was no mineral incorporation in the Ob nodule but abundant minerals in the nodules containing lacZ^+^ green/blue Ocys (**Figure** 3h).

Together, the comprehensive data indicate that Ocys, but not Obs, deposit minerals surrounding cell bodies and dendrites to entire bone matrices (including the bone surface), in contrast to the common belief that minerals are added from the outside by Obs.

### Blood Vessels in Cortical Bones Are Embraced by Numerous Osteocytes and Their Dendrites

**Figure 4:**
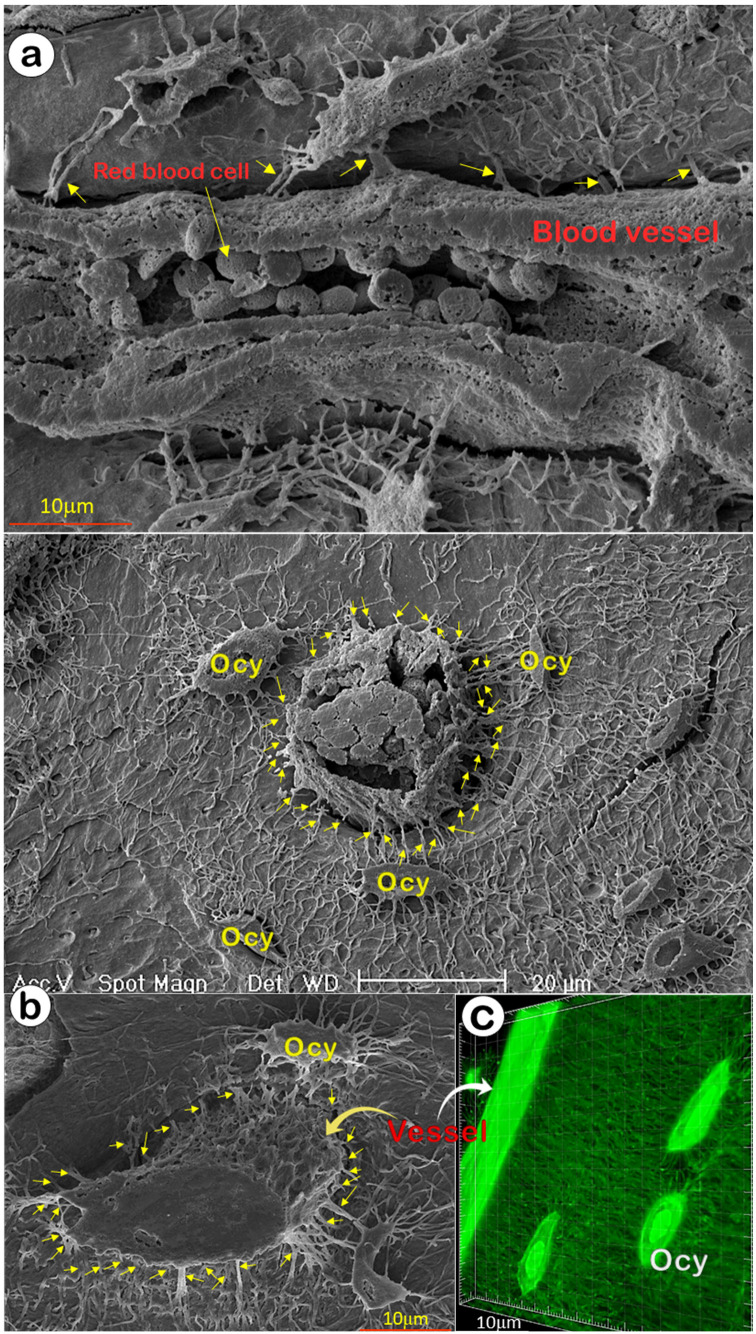
** Direct communication between vessels and Ocy-dendrites** (Representative data from 2-4 animals for each assay)**.** (a) The mouse long bone acid-etched SEM images revealed that a medium-size vessel (sagittal view with numerous red blood cells) was enclosed by Ocy and its dendrites (arrows). (b) A representative small vessel (cross view; upper panel) with numerous red blood cells, and a large vessel (cross view, lower panel) connected with numerous Ocys and their dendrites (arrows). (c) A confocal view of a mouse long bone vessel with numerous Ocy dendrites (arrows).

The ability of OCYs to form mineralized bone depends on the appropriate transfer of oxygen and nutrients from circulation. It is widely assumed that mineralized bone has few blood vessels and that the canalicular fluid outside Ocys transports nutrients 14, although recent studies indicated a direct connection between vessels and Ocys 45, 46. In this study, we used acid-etched SEM images from a one-month-old mouse cortical bone (with two separate views: the sagittal, **Figure** 4a; and the cross-view, **Figure** 4b), and FITC-stained non-decalcified mouse femoral bone (**Figure** 4c) that showed direct evidence of rich blood vessels in bone matrices; there were dense connections between blood vessels and Ocys, revealing a rough vessel architecture instead of the smooth surface commonly presented in textbooks.

### A Drastic Increase in Osteoblast-Like Cells in *Dmp1* Null Mice (A Hypophosphatemia Rickets Model)

**Figure 5:**
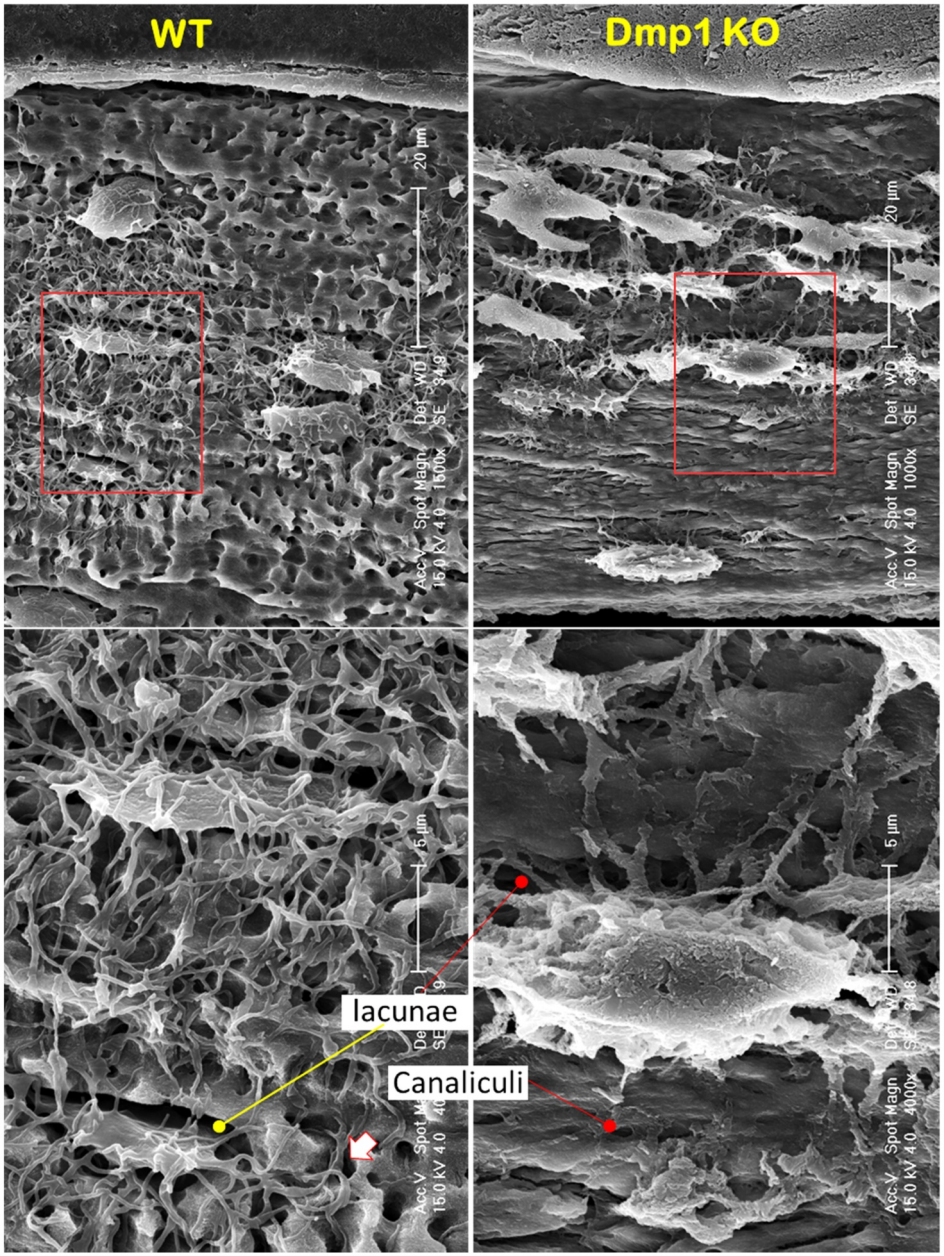
** A loss of the “wire mesh” structure and a change of Ocy morphologies in the *Dmp1* null mandible (**Representative data from 4 animals**)**. Acid-etched SEM images from a 7-week-old mandible bone revealed a “rod and wire mesh-like” structure in the control sample, which maintained an intact bone structure (left, n = 4). In contrast, the age-matched *Dmp1* KO bone displayed a poorly formed mandibular bone, in which the “rod and wire mesh-like” structure was largely disappeared with striking changes in Ocy morphologies, including more cell numbers, an overall increase in cell size, a switch from spindle to olive shape, an increase in dendrite diameter, and a decrease in dendrite length.

**Figure 6:**
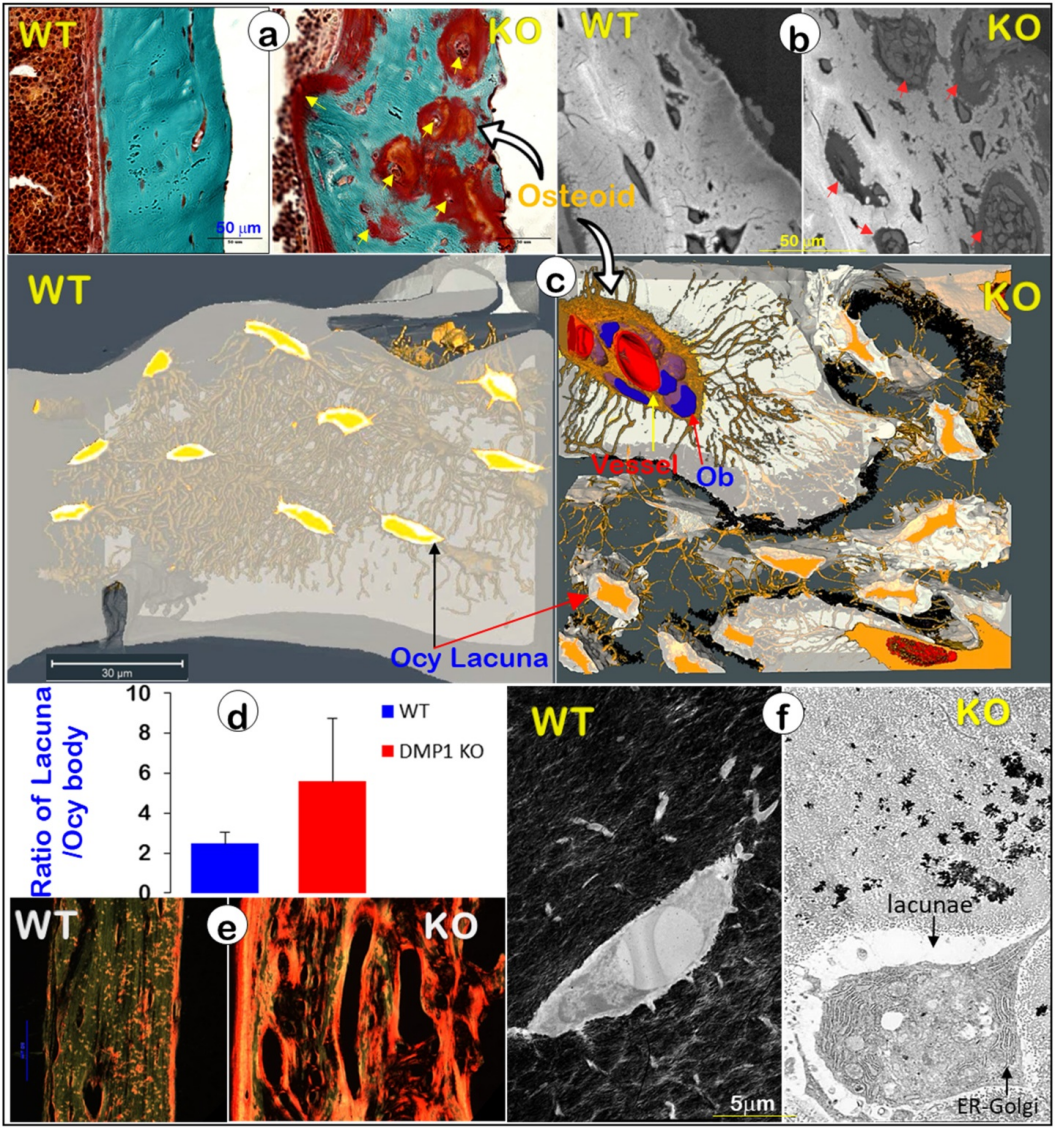
** Blocking osteocyte maturation leads to poor mineralization in the *Dmp1* KO long bone (right panels)** (Representative data from 2-4 animals for each assay). (a). Goldner stain images displayed a sharp increase in osteoid thickness on bone surfaces and many osteoid clusters in the *Dmp1* KO long bone (arrows). (b). Backscattered SEM images revealed numerous OB-like cells in these clusters (arrows). (c). VolumeScope imaging data showed the nature of OB-like cells in the clusters plus the enlarged Ocy lacunae in the *Dmp1* KO mice. (d). Quantitative data demonstrated a significant increase in the ratio of lacuna/Ocy cell bodies in the* Dmp1* KO compared to the control (n = 9-10; p < 0.05). (e). The Sirius Red images under polarized light microscope exhibited an increase in collagen type I in the* Dmp1* KO bone. (f). The non-decalcified TEM images displayed a sharp reduction in mineralization plus an increase in the Ocy lacunae of the* Dmp1* KO bone.

It is widely believed that a defect in Ob function is responsible for bone diseases such as rickets/osteomalacia (a defect in mineralization), although strong evidence supports the vital role of Ocys in mineralization 20. To better define the cell type (Obs or Ocys) that is responsible for the onset of mineralization defects in rickets, we reinvestigated *Dmp1* gene (highly expressed in Ocys) 47, 48, and *Dmp1*-knockout (KO) mice, in which Obs fail to form Ocys 44. Using acid-etched SEM images, we revealed drastic changes in bone structures and cellular morphologies in a 7-week-old *Dmp1* KO mandible bone (**Figure** 5, right). These changes included increases in cell numbers and cell size, a switch from a spindle to an olive shape, and an increase in dendrite diameter but a sharp decrease in dendrite length, as well as a largely disappeared “ rod and wire mesh-like” structure, compared to the age-matched control (**Figure** 5, left). In histological studies, we found numerous Ob-like cells buried in the osteoid of the null mouse bone matrices (including surfaces and deep bone) with no sign of peri-cellular mineral deposition by Goldner staining and backscattered SEM (**Figure**. 6a-b). VolumeScope images further confirmed the nature of Ob-like cells in the cluster, in which there were even a few small vessels (**Figure**. 6c, right; **Movie**s S2-3). In addition, the KO Ocy lacunae were greatly enlarged in comparison with the age-matched control (**Figure**. 6c, left), which is statistically significant (**Figure**. 6d). The Sirius Red staining images under polarized light displayed abundant collagen (a parameter to reflect Ob function) in the *Dmp1* KO bone (**Figure**. 6e, right). The non-decalcified TEM images showed a cuboid-like (instead of spindle-shaped) Ocy in *Dmp1* KO mice with extensive Golgi-apparatuses that had few dendrites and little minerals in the matrix (**Figure**. 6f).

**Figure 7:**
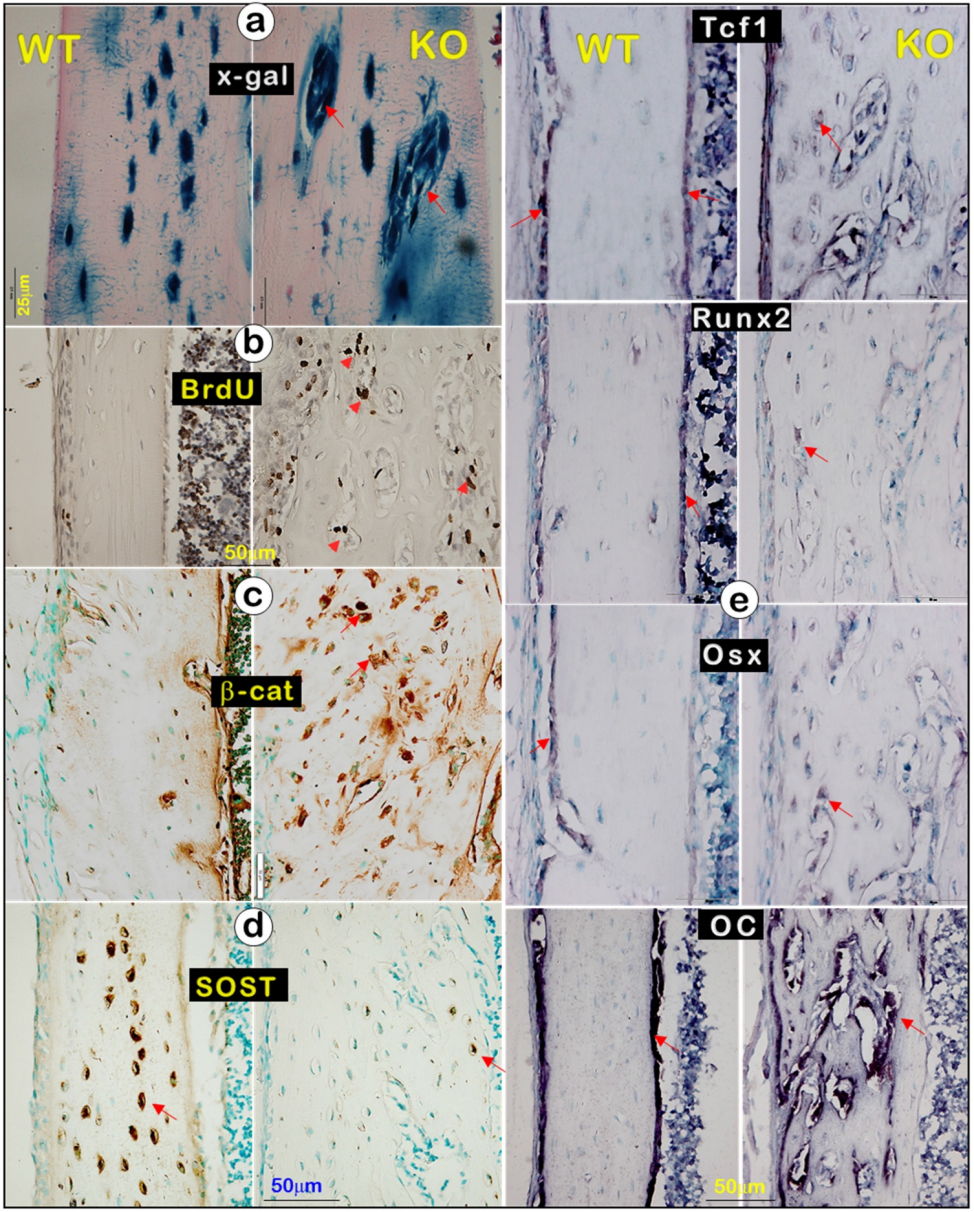
** A great decrease in mature Ocy marker (SOST) but a sharp increase in osteoblast markers in the *Dmp1* KO long bone (right panels) (**Representative data from 3 animals at age of 3 weeks**).** (a). X-gal stain images displayed *Dmp1*-LacZ^+^ Obs (instead of Ocys) (arrows). (b). BrdU staining revealed a sharp increase in cell proliferation in the* Dmp1* KO long bone. (c). β-catenin immunostaining showed a great increase in β-catenin expression in the *Dmp1* KO bone. (d). SOST immunostaining showed a dramatic reduction in SOST level in the *Dmp1* KO bone compared to the control. (e). *In situ* hybridization images exhibited an increase in Ob markers (Tcf1, Runx2, Osx, and OC) in the *Dmp1* KO bone matrix compared to the control surface.

In studies of molecular mechanisms, we showed many *Dmp1*-*lacZ^+^* cells in the clusters via x-gal staining (**Figure**. 7a, right), an increase in BrdU (**Figure**. 7b, right) and β-catenin (**Figure**. 7c, right), along with a sharp decrease in SOST (a strong antagonist of Wnt-β-catenin 49, 50; **Figure**. 7d, right), reflecting an enhanced Ob function in *Dmp1*- KO mice compared to the WT mice. To further test this hypothesis, we performed *in situ* hybridization on tibias at the age of 3 weeks using the following Ob markers: *Tcf1*, *Runx2*, *Osx*, and osteocalcin (OC). In the WT bone, these markers were mainly detected in the surface Ob layer (**Figure** 7e, left). In contrast, they were expressed in entire bone cells in* Dmp1* KO mice (**Figure** 7e, right), indicating that the KO bone is filled with Ob-like cells with an increased Ob activity, instead of a decrease in Ob activity as commonly believed.

### Hyp Mice Display An Identical Phenotype to That of *Dmp1*-KO Mice with Sharply Increased β-catenin Activity

**Figure 8:**
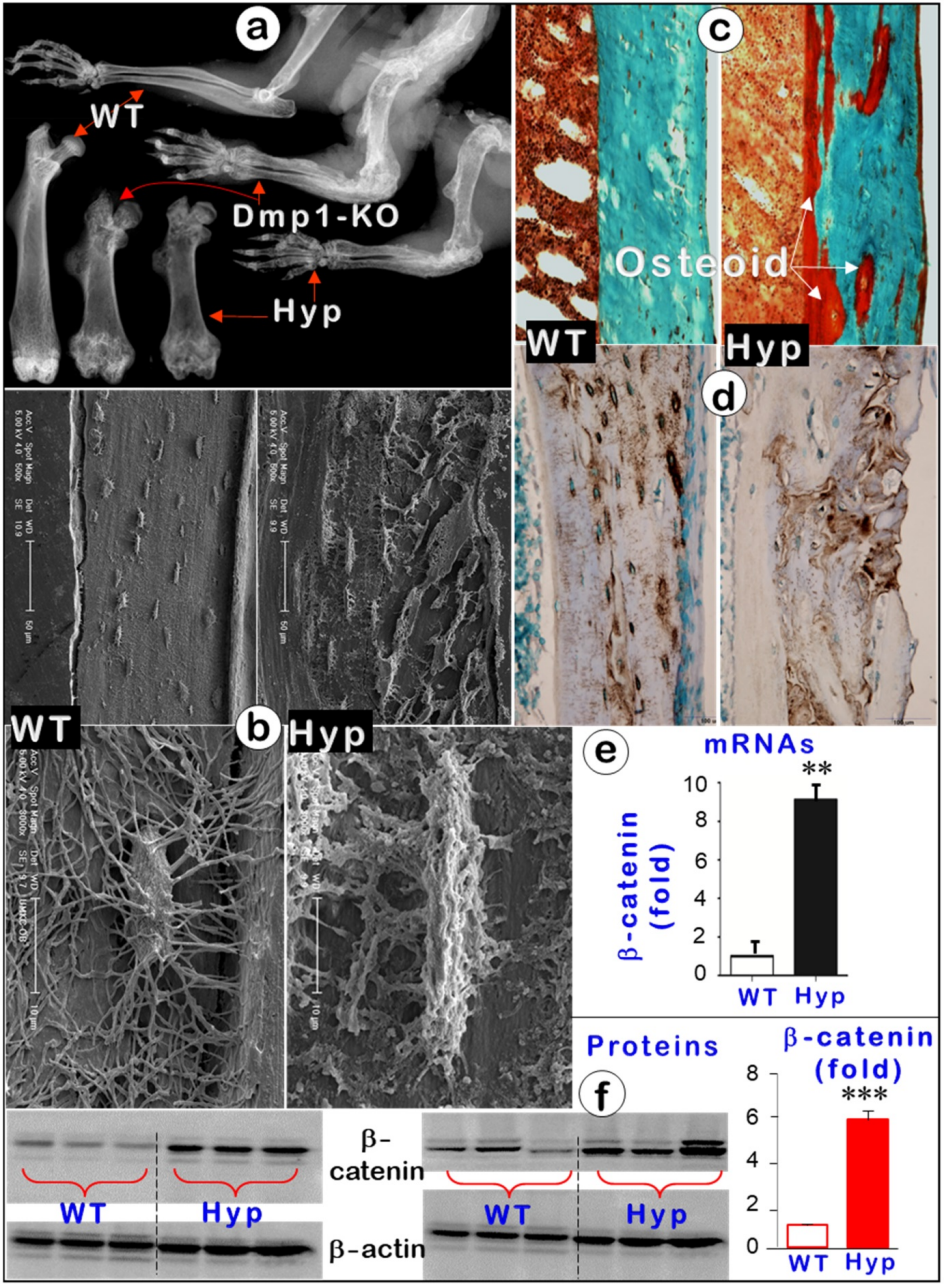
** Hyp mice displayed an identical osteomalacia phenotype as that for the *Dmp1* KO long bone** (Representative data from 3-6 animals for each assay). (a). X-ray images displayed very similar changes in osteomalacia long bones from Hyp and *Dmp1*-mice. (b). Acid-etched SEM images showed a great increase in osteoid within the Hyp long bone (arrow) plus a defect in Ocy morphologies (an increase in cell body and dendrite thickness and a rough surface). (c). Goldner staining showed a great increase in osteoid in the Hyp bone. (d). DMP1 immunostaining revealed no apparent change in DMP1 expression level except for a pattern change in the Hyp bone. (e-f). Quantitative data displayed a significant increase in b-catenin in Hyp mRNAs (e, n = 6; p < 0.01) and proteins (f, n = 6; p < 0.001) in Hyp long bones compared to the age matched wild type long bones.

To further support the key role of Ocys (instead of Obs) in mineralization, we also studied Hyp mice, a commonly used osteomalacia model due to their *Phex* mutation 51, 52. These mutant mice displayed an osteomalacia phenotype identical to that of *Dmp1*-KO mice. Features included short limbs with flaring joints (**Figure** 8a), large amounts of osteoid, and malformed Ocys (**Figure** 8b-c, right) in the absence of apparent changes in DMP1 levels (**Figure** 8d, right). In mechanism studies, we showed that there was a significant increase in β-catenin levels of mRNAs (around 10-fold; **Figure** 8e) and proteins (6-fold; **Figure** 8f) in the Hyp mice, suggesting the likely pathological role of β-catenin in the onset of osteomalacia.

### An Increase in β-catenin in Osteocytes Recaptures the *Osteomalacia* Phenotype

**Figure 9:**
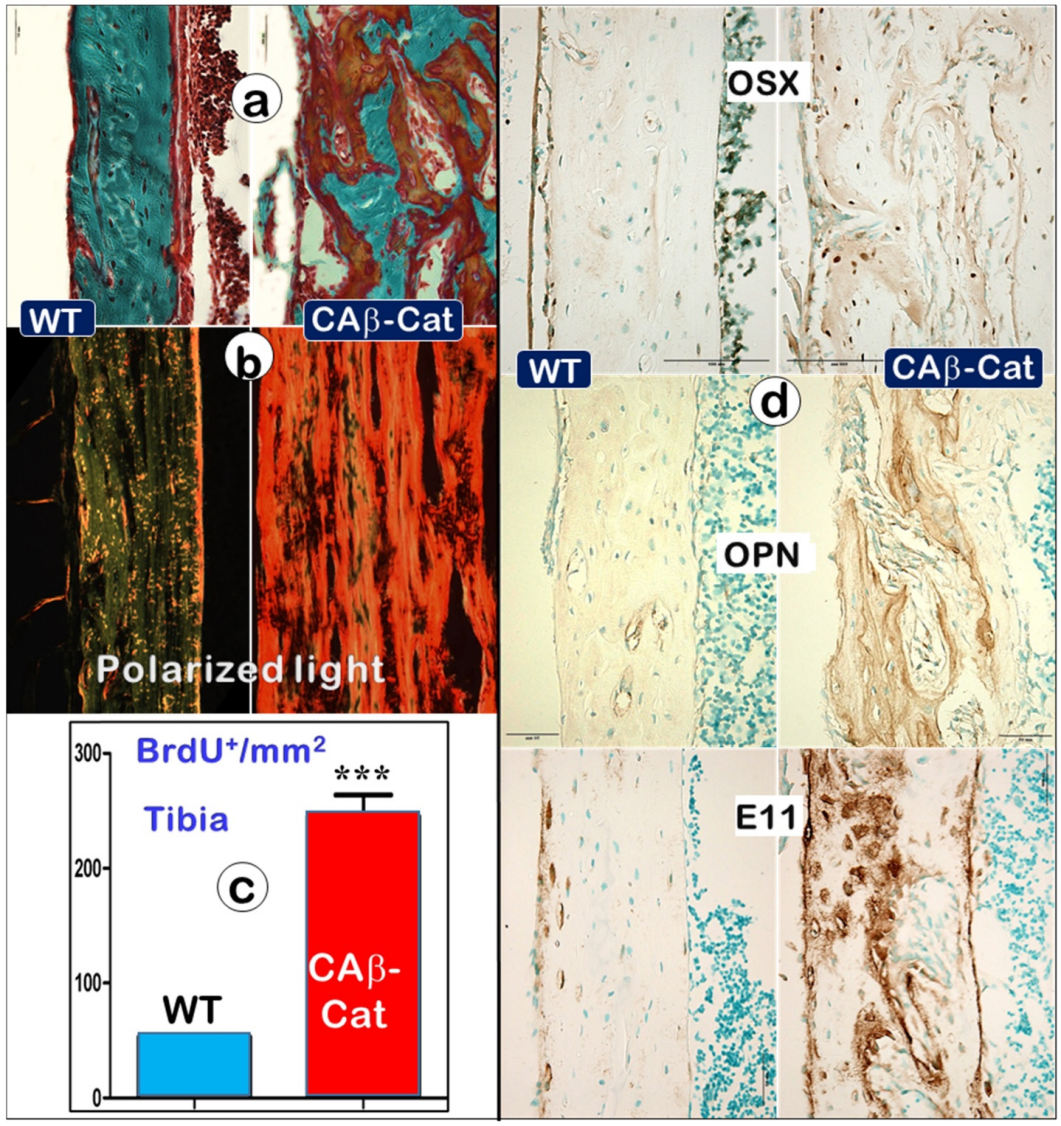
** An increase in b-catenin within Ocys (CA-β-Cat, constitutive stabilization of β-catenin) recaptures the *Dmp1* KO long bone phenotype (right panels) compared to the control (**Representative data from 4 animals at the age of 4 weeks**).** (a). Goldner stain images showed a great increase in osteoid in the CA-β-Cat long bone. (b). The polarized microscope images exhibited an increase in collagen type I in the CA-β-Cat long bone. (c). BrdU staining revealed a sharp increase in cell proliferation within the CA-β-Cat long bone (n = 4; p < 0.001). (d). Immunostaining images revealed a great increase in bone markers within the CA-β-Cat long bone, including OSX, OPN, and E11.

To test the above hypothesis, we constitutively stabilized β-catenin (CA-β-Cat) in Ocys by crossing *Dmp1*-Cre 22 to CA-β-Cat 24 mice. The increased CA-β-Cat level in osteocytes led to an osteomalacia phenotype very similar as observed in *Dmp1* KO and Hyp mice, including a great increase in osteoid within the entire long bone (**Figure** 9a, right), high levels of type I collagen production (**Figure** 9b, right), an increase in BrdU^+^ cells (**Figure** 9c, right), and drastic increments of Ob markers such as OSX, OPN, and E11 (**Figure** 9d, right). Together, these data support the notion that the increase of CA-β-Cat in Ocys (the key causative factor in hypophosphatemia rickets) maintains high Ob activity, which is directly linked to defects in mineralization.

## Discussion

Although Ocys are derived from the surface Obs 53, these long-living cells (with enormous cell surfaces embedded in bone matrices in very large cell numbers) have been largely overlooked in studies of bone biology and the onset of bone diseases such as rickets. In this study, we used multiple imaging techniques plus three genetic mouse models to test the hypothesis that Ocys play a vital role in bone mineralization and the onset of hypophosphatemia rickets. Our key findings are that Ocys (not OBs) build bone in ways similar to the construction of a high-rise building, with a “wire mesh-like” frame (i.e., Ocy dendrites) and cement (mineral matrices secreted from Ocys). When Obs fail to differentiate into Ocys in *Dmp*-1-null or Hyp (a Phex mutation) mice during development, aberrant and poor bone mineralization occurs due to a sharp increase in Wnt-β-catenin signaling, in which there is a high (instead of low) Ob activity.

For over a century, Obs have been viewed as the cells responsible for bone formation 54, 55. In the last decades, more and more evidence has greatly challenged this view due to applications of cutting-edge technologies such as cellular and molecular biology, genetically engineered mouse models, mutation case reports, and advanced imaging technique. New information suggests that the third bone cell (Ocy, the descendant of Ob) plays broad roles 55 56, including those in mineralization. For example, Baylink and Wergedal first showed the *in vivo* evidence that Ocys form bone, although they concluded that Ocys do not play a major role in comparison with Obs 57. Later, Barragan-Adjemian et al. raised an appealing hypothesis that Ocys form calcified spherical structures using an Ocy cell line 58. However, the old view remains dominant: *“Osteoblasts are one of the three cell types found in vertebrate bones. Osteoblasts synthesize the bone collagen matrix of the bone and also participate in matrix mineralization, which provides strength. When osteoblasts are trapped into the matrix, they become osteocytes. While it is formed by osteoblasts, the bone matrix is degraded by osteoclasts*” (https://www.nature.com/subjects/osteoblasts). In this study, we challenged this view in the following 5 aspects.

First, similar to building a house, it is impossible for a builder to finish this project without working inside the house (i.e., the surface Obs are not in the right position to do this job). Instead, the entire bone is built by Ocy dendrites, similar to constructing a high-rise building with a “wire mesh-like” structure (**Figure** 1; **Figure** 5, left). Second, bone mineralization is a slow and lengthy process (**Figure** 2), while Obs with a very short life span 59 are not fit for this function. Third, mineralization directly takes place surrounding Ocy dendrites, leading to strong mineral rings distributed around Ocy dendrites but not Obs (**Figure** 3; **Figure**s S2-3). Remarkably, minerals on bone surfaces and bone vessels are also produced from deep Ocy dendrites (but not from OBs), supporting the mineralization direction from inside to outside but not from outside to inside (a common belief, **Figure** 3; **Figure**s S2-3). Fourth, there are plenty of vessels in bone matrices, which are completely wrapped by Ocy dendrites (**Figure** 4), indicating an efficient blood-Ocy-dendrite network for nutrition/minerals moving in and out. Because of this massive connection network, the vessel surface in bone matrices is not smooth as commonly believed but is rough. Fifth, *Dmp1* KO mice displayed a largely destroyed “wire mesh-like” bone structure (**Figure** 5) with widely distributed osteoid on both bone surfaces and deep bone due to loss of Ocy functions (**Figure** 6). At the cellular level, we showed Ob-like cells within the osteoid with richness of collagens in bone matrices (**Figure** 6). Furthermore, two additional rickets models derived from Hyp mice (**Figure** 8) and targeted stabilization of β-catenin in Ocys (**Figure** 9) showed a very similar osteomalacia phenotype due to a sharp increase in β-catenin in Ocys (i.e., the causative factor for the onset of rickets); this event led to an increase in Ob-like cell numbers and activities but a decrease in mature Ocy numbers and their activities.

The widely accepted concept that Obs form bone was established at a time when modern imaging technologies were not available. Ocys, derived from Obs but buried within the bone, were largely treated as “retired bone builders” partly due to technical difficulties in visualizing them and partly due to their longevity in studies of their roles in bone biology. This circumstance has now changed, and studies on the roles of Ocys in bone health and disease are now possible, as demonstrated by the experiments in the current study, in which we developed several new research tools and modified a number of old assays for studies of mineralized bone. For example, in defining cells that are truly responsible for mineral deposition, we used a confocal microscope (providing high imaging quality for weak signals) with DAPI staining (displaying cells that deposit minerals), which unveiled a detailed mineralization process at the cellular level (showing double labeling lines around Ocys that correlated with the same labeled lines on bone surfaces (**Figure** 3a). We also combined a one-time injection of Alizarin Red in 2.3 Col 1 GFP mice (in which both Obs and Ocys are in green color) by confocal imaging plus backscattered SEM to define the direct role of Ocys in the formation of the strong mineral rings around them (**Figure** 3d-e). To define the “wire mesh structures” in bone, we combined the acid-etched SEM, backscattered SEM, and TEM methods for better visualization of these structures (**Figure** 1). Furthermore, we combined a classic mineralization assay (Von Kossa stain) and backscattered SEM to show a direct link of mineralization and Ocys (but not Obs; **Figure** 2). Notably, we introduced VolumeScope (a new imaging tool used in soft tissues such as a nerve system) into bone biology. This addition not only helped define the difference between the newly formed Ocys on the bone surface and the mature Ocys in the deep bone at EM levels (**Figure** 2g and **Movie** S2), but also revealed the nature of bone cells (i.e., Ob-like cells and vessels) in the osteoid cluster from a rickets bone specimen (**Figure** 6 and** Movie** S3).

**Figure 10:**
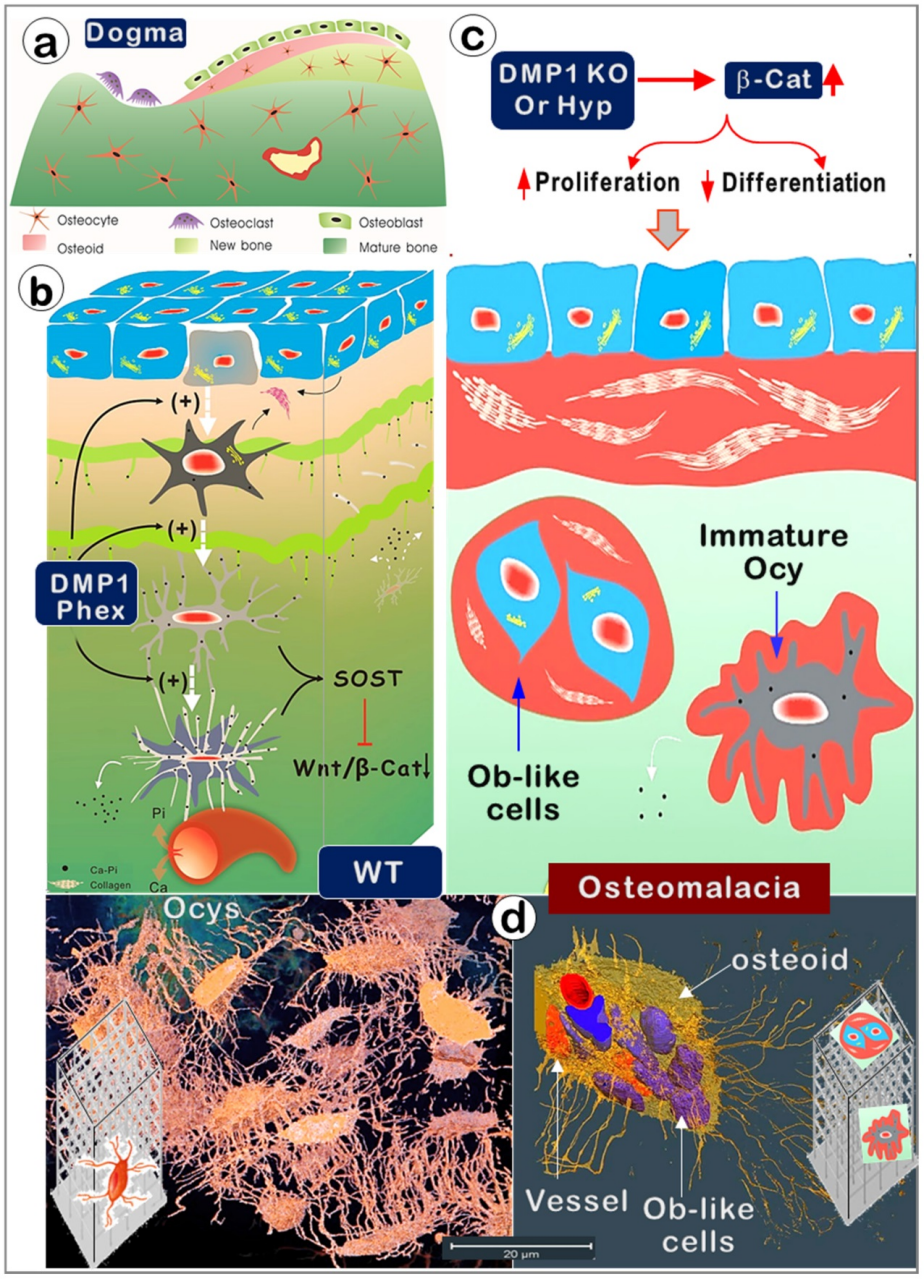
** Old versus new views regarding cells responsible for bone mineralization and the onset of rickets.** (a). The prevailing theory in bone biology is that new bone is formed by osteoblasts (Obs) from the bone surface. (b). The new model (that Ocys form mineralized bone) includes the following four key components. **i**). Nascent Ocys (dark grey) produce collagen matrixes and small amounts of mineral (Ca-Pi) surrounding the “wire-mesh” built by Ocys-dendrites. **ii**). Working together, the well-formed Ocys (light grey) and the mature Ocys (white) deposit Ca-Pi in the *bone matrices*, “pump” Ca-Pi to the *bone surfaces*(*two green lines with numerous “thorns” indicating the mineral that originated from the inner Ocys),* and fill in the early formed *Ocy space*(blue). This space is greatly depleted during the long, slow bone maturation process; **iii**). Ocys directly take Ca, Pi, and nutrients from the blood vessels; **iv**). DMP1 and Phex are the key molecules controlling the differentiation of Obs to Ocys and the subsequent Ocy maturation; **v**) The matured Ocys produce SOST (sclerostin, a potent inhibitor of Wnt-β-catenin) to maintain Wnt signaling at a low level. (c). In the *Dmp1*-null (KO) or Phex mutation (Hyp) mice, the immature bone cells release large amounts of β-catenin (β-Cat), which accelerates cell proliferation but inhibits cell differentiation. As a result, the defective bone cells that are arrested either at the late Ob stage (although preserving high Ob activity; blue cells) or at the immature Ocy stage (grey cells) fail to “pump” enough Ca-Pi to the surrounding bone matrices and bone surfaces (red, osteoid; green, mineralized matrices), leading to osteomalacia. (d). The real 3d view of healthy OCYs (left) versus Ob-like cells in rickets bone (right) by use of a newly established VolumeScope technique.

In summary (**Figure** 10), this study challenges the Obs forming bone theory (the cornerstone of bone biology for over a century) by introducing and modifying numerous *in vivo* and *in vitro* methods. Our studies demonstrate that Ocys directly form mineralized bone during development. The data presented also reveal that defects in Ocys lead to the onset of osteomalacia via a sharp increase in β-catenin mechanisms. The methods developed will help future studies of bone formation in health and disease.

## Supplementary Material

Supplementary figures and video legends.

Supplementary video 1.

Supplementary video 2.

Supplementary video 3.
