# Supplementary material for: Osteocytes but not osteoblasts directly build mineralized bone structures
Source: Int J Biol Sci. 2021 Jun 11;17(10):2430–48. doi: 10.7150/ijbs.61012 (PMC8315029; doi:10.7150/ijbs.61012)
Supplement: Supplementary file 1 — Supplementary figures and video legends. [file ijbsv17p2430s1.pdf]

Supplementary Figures

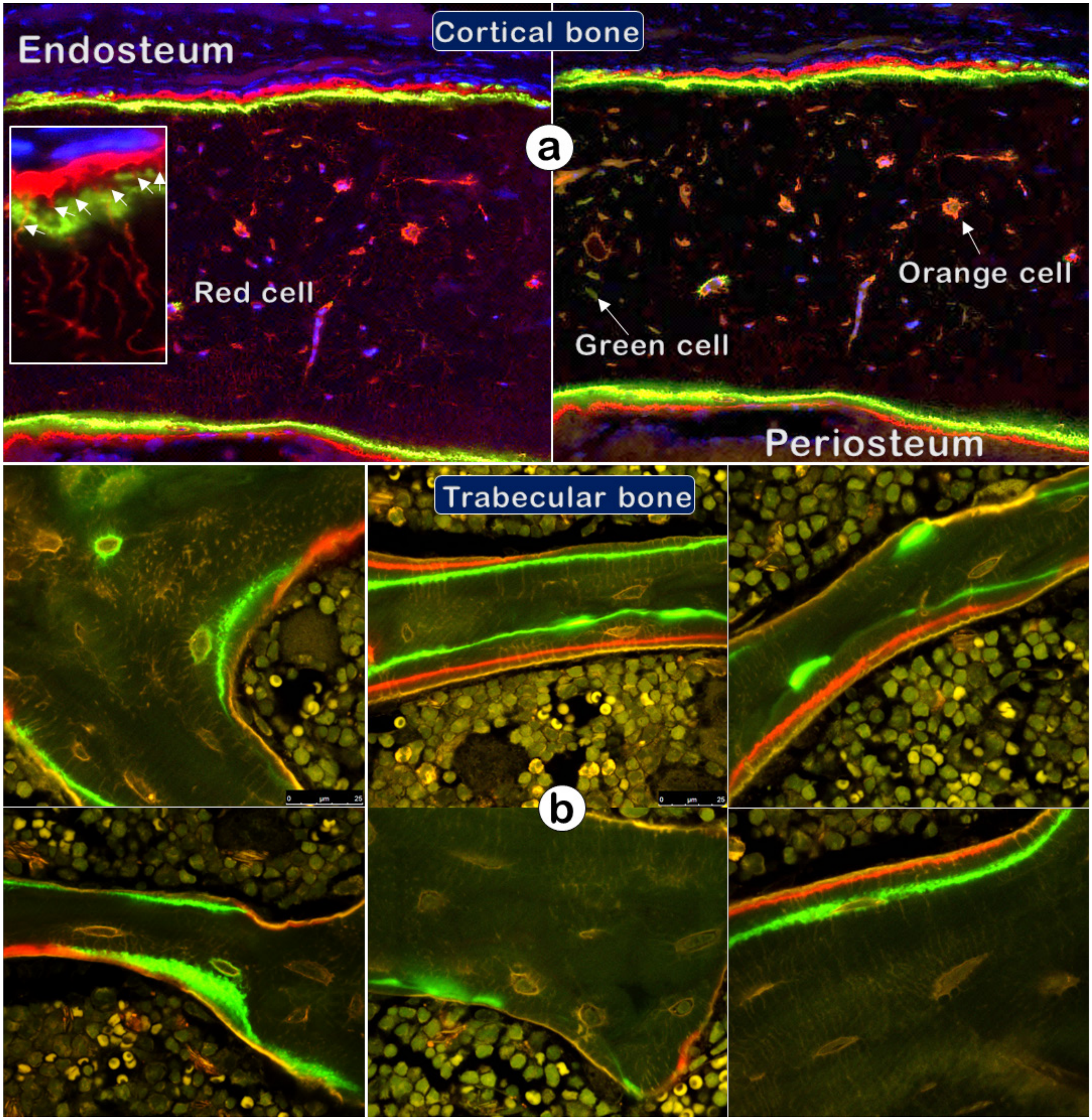

Supplementary Figure 1. Minerals grow from the inside to the outside with diversified deposition patterns (Representative data from 4 animals at age of 7-8 weeks**).**

Calcein was injected into 7-wk-old WT mice followed by Alizarin Red 5 days later. The mice were sacrificed at the age of 8 wks. A series of 10-mm sections from a plastic-embedded long bone was counter-stained with DAPI followed by confocal imaging.

(a). The two sagittal sections displayed double-labeled mineralized fronts with red-stained osteocytes and their dendrites (surface; left), and orange and green osteocytes (deep; right), suggesting that osteocytes continuously deposit minerals in bone matrices and bone surfaces (insert).

(b). The enlarged trabecular bone sections showed a yellow line (reflecting an osteoid layer) plus numerous yellow osteocytes on the top of green and red lines as well as green osteocytes. This finding indicated 1. a small amount of minerals in osteoid layers are likely derived from inside osteocytes; 2. a constant mineral deposition by osteocytes (i.e., green and red labels giving rise to yellow color).

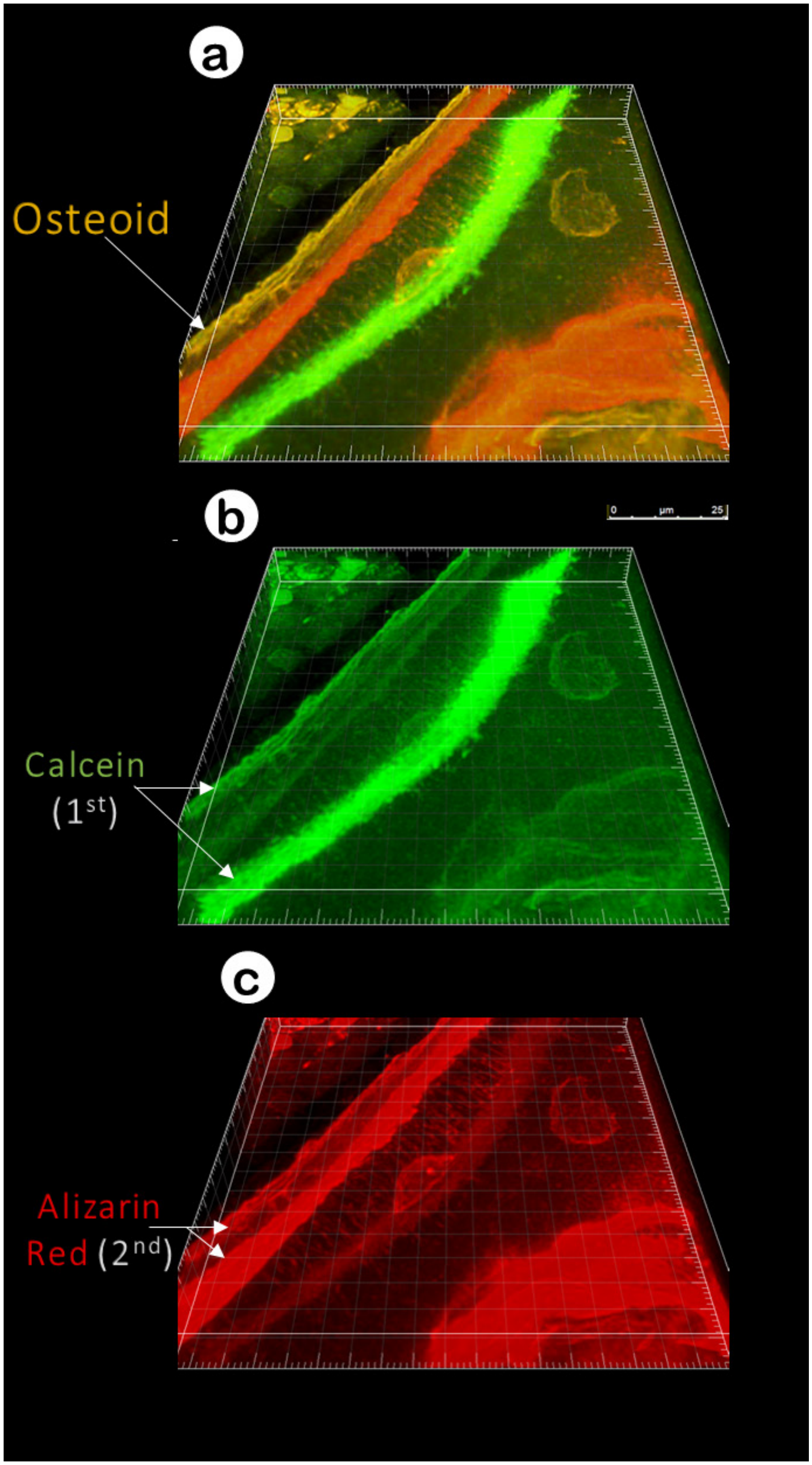

Figure S2. The deep osteocytes contribute to mineral content in the osteoid layer
(There is a small amount of minerals in the osteoid layer, which is mainly formed from inner Ocys**.** Calcein was injected into 7-wk-old WT mice followed by Alizarin Red 5 days later. The mice were sacrificed at the age of 8 wks).
(a). Overlapping both red and green lights revealed an orange line, reflecting contributions from inner Ocys for a small amount of minerals in the osteoid layer.
(b). The green light view only, showing one bold line and one thin surface line.
(c). The red light view only, showing one bold line and one thin surface line.

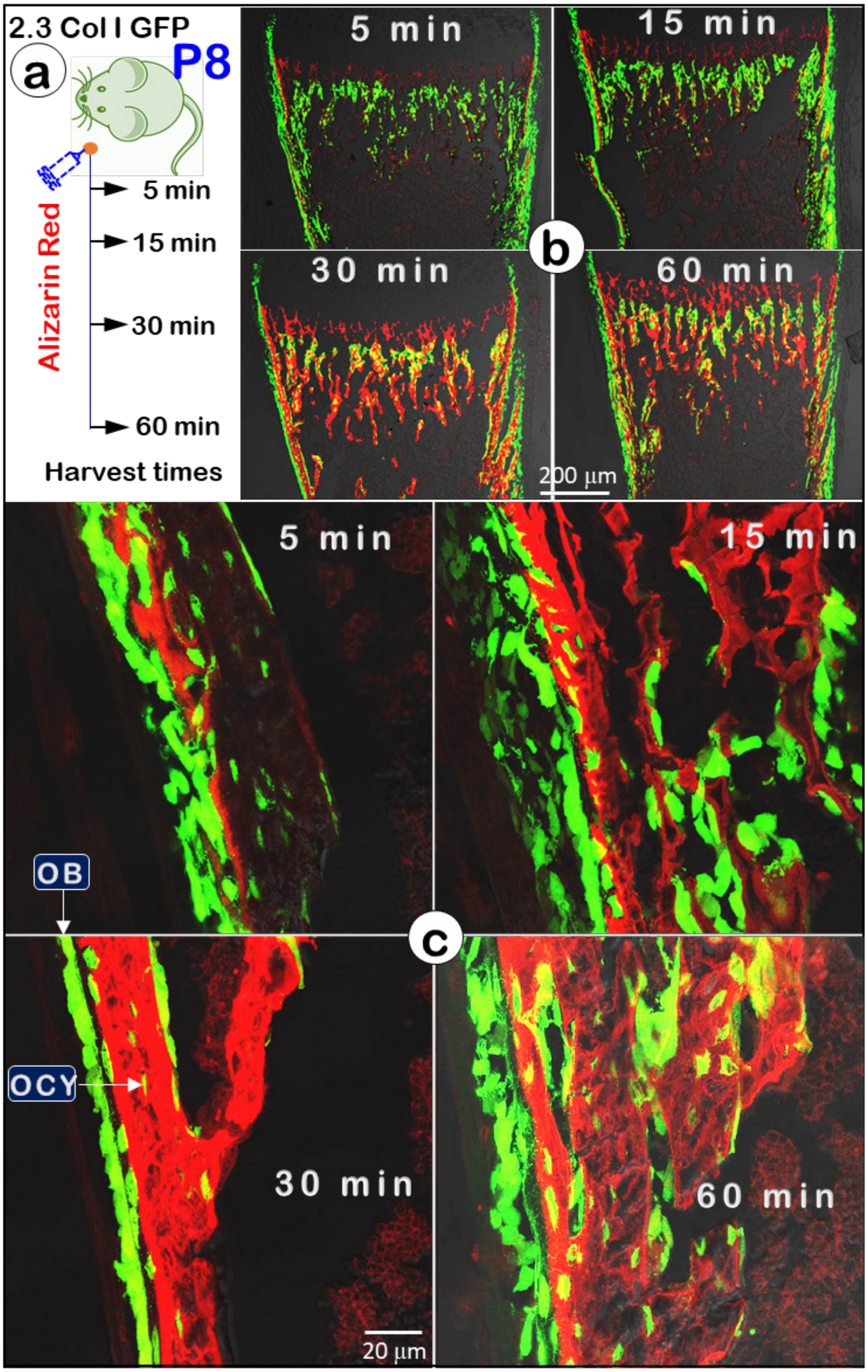

Figure S3. The early mineral deposition contributed by OCYs but not OBs in a 2.3 Col1-GFP mouse model (Representative data from 4 animals at the age of P8**).**

(a). Schematic diagram describing a one-time Alizarin Red i.p. injection at postnatal day 8 (P8) in 2.3 Col 1-GFP pups with samples collected at 5 min, 15 min, 30 min, or 60 min after injection, respectively.

(b) Low magnification images of mineral deposition displayed a gradual increase in Alizarin Red staining in bone matrices.

(c) Enlarged magnification views revealed a lack of minerals in areas surrounding an Ob lining layer, whereas the red-stained mineral is adjacent to OCYs but not to Obs.

Movie S1. Enlarged double labeling in an adult mouse long bone. The movie displayed the sole contribution of Ocy and its dendrites for mineralization (Calcein, 1st; Alizarin Red, 2nd; 5 days apart).

Movie S2. A 2-month-old mouse long bone with the VolumeScope technique.

Movie S3. A 2-month-old Dmp1 KO long bone with the VolumeScope technique.
